# Screening factors to affect ultrasound-assisted extraction of (poly)phenols from date palm seeds

**DOI:** 10.3389/fchem.2024.1409393

**Published:** 2024-07-16

**Authors:** Raquel Lucas-González, Manuel Viuda-Martos, José Ángel Pérez-Álvarez, Juana Fernández-López

**Affiliations:** Institute for Agri-Food and Agri-Environmental Research and Innovation, Miguel Hernández University (CIAGRO-UMH), Alicante, Spain

**Keywords:** sonotrode, (poly)phenols, Plackett–Burman design, soluble-conjugate, ultrasound-assisted extraction

## Abstract

The aim of the current work was to compare the (poly)phenol profile (free, soluble-conjugate, and insoluble-bound) and antioxidant activity of date palm seed flour using different extraction methods (conventional vs. ultrasound-assisted extraction [UAE]) and to determine the most critical variables in the extraction of (poly)phenols through UAE using the Plackett–Burman design experiment. Using the Plackett–Burman design, seven factors, namely, ethanol concentration, liquid:solid ratio (mL/g), sonotrode, amplitude (%), extraction time, extractant pH, and extraction cycle, were studied. After the factors were studied using conventional extraction methods, 23 compounds were quantified, with protocatechuic acid and catechin being the predominant (poly)phenols. Furthermore, the distribution of (poly)phenols within the cell varied, with glycosylated quercetins and caffeoyl shikimic acids predominantly found in free forms. Ultrasound-assisted extraction demonstrated efficiency in extracting free and soluble-conjugate (poly)phenols. However, it showed limitations in extracting insoluble-bound (poly)phenols. Nevertheless, similar amounts of total (poly)phenols were shown after conventional extraction and UAE, that is, 259.69 ± 43.54 and 189.00 ± 3.08 mg/100 g date seed flour, respectively. The Plackett–Burman design revealed the liquid–solid ratio as a crucial factor affecting (poly)phenol extraction, with higher ratios yielding better results. The sonotrode choice also influenced the extraction efficiency, highlighting that the sonotrode with a smaller diameter but higher displacement amplitude showed the best polyphenol recovery and antioxidant activity values. The nature of (poly)phenols influenced the studied extraction variables differently, emphasizing the complexity of the extraction process. In this line, pure water was sufficient to extract flavan-3-ols after UAE, whereas ethanol was a crucial factor in extracting quercetin. These findings underscore the importance of optimizing extraction methods for maximizing (poly)phenol recovery from date palm seed flour for various applications in food and pharmacology industries.

## 1 Introduction

The date fruit of the date palm (*Phoenix dactylifera* L.) is a product of great cultural, social, and economic importance in many countries, having special relevance in arid regions (Fernández-López et al., 2022). The main co-product generated during the industrialization of dates is the date seed, representing approximately 10% of the fruit weight. Considering that the world date production in 2022/2023 was 9.75 million tons (Shahbandeh, 2024), date seed production will reach approximately 975,000 tons, with most of them being discarded or used as fodder for domestic farm animals. However, date seeds are a rich source of dietary fiber, mainly insoluble dietary fiber, whose values range from 50% to 70% (Afrazeh et al., 2021; Kamal et al., 2023), and (poly)phenols, whose values range from 1,864 to 4,768 mg gallic acid equivalent/100 g. Flavan-3-ols, especially catechins and epicatechins, are the most abundant group, followed by phenolic acids, such as protocatechuic acid, *p*-hydroxybenzoic acid, and caffeoyl shikimic acid (Al-Farsi and Lee, 2008; Muñoz-Tebar et al., 2024). Therefore, in line with the Sustainable Development Goals and the 2030 Agenda, the valorization of food co-products has been the subject of the scientific community in recent years. One strategy to valorize food co-products is to obtain phytochemical compounds for use as nature food additives, to enrich foods, or to be applied in the cosmetic and pharmaceutical fields. Although several extraction technologies could be applied, the use of green and emerging technologies, such as ultrasound-assisted extraction (UAE), pulsed electric field (PEF), accelerated solvent extraction (ASE), supercritical fluid extraction (SFE), or microwave-assisted extraction (MAE), instead of conventional methods (using organic solvents), is proving to be much more appropriate because in addition to contributing to the care of the environment by the application of less polluting and more environmentally friendly processes, they would also improve the extraction efficiency. In this way, UAE has been revealed as one of the most efficient techniques for the extraction of phenolic and antioxidant compounds from agro-industrial co-products (Barba et al., 2016; Kumar et al., 2021). Green and emerging technologies enhance not only the extraction yield (total phenolic and total flavonoid content) but also the antioxidant capacity of the extracts compared with conventional extraction techniques (Patil et al., 2022; Paul et al., 2023). However, optimizing UAE conditions is necessary to minimize the extraction time, solvent consumption, and costs while maximizing efficiency to achieve extracts rich in bioactive compounds with high purity. Moreover, the designs of experiments, such as factorial designs and Plackett–Burman, for screening multiple factors efficiently, or Box–Behnken design, for exploring the intricate relationships between variables, are considered good strategies for carrying out optimization processes (Anaya-Esparza et al., 2023).

This aim of this work was to evaluate the effectiveness of ultrasound-assisted extraction (probe-type sonication) to extract (poly)phenols from date palm seeds and to identify the most critical variables in the UAE using the Plackett–Burman design (PBD). The effectivity of UAE was evaluated by comparing the polyphenol profile obtained by UAE with that obtained using the conventional extraction methods used to determine the three (poly)phenol fractions in the date palm seed: free, soluble-conjugate, and insoluble-bound.

## 2 Materials and methods

### 2.1 Plant material

Date (*P. dactylifera* L. cv. Medjool) seed flour with a particle size <0.210 mm was obtained following the method previously described by Muñoz-Tebar et al. (2024). A total of 300 g of date seed flour, previously dehydrated (48 h at 55 °C), ground (stone mill), and sifted, was obtained.

### 2.2 (Poly)phenol extraction

Three conventional extraction methods were used to obtain the free, soluble-conjugate, and insoluble-bound polyphenols from date palm seeds. The free and insoluble-bound (poly)phenols were extracted as previously described by Lucas-González et al. (2023), but with slight modifications. Specifically, the incubation period in the insoluble-bound extraction was improved with agitation, as described below.

#### 2.2.1 Free (poly)phenols

A measure of 3 g of the sample was mixed with 30 mL of aqueous methanol (20:80 v/v) (solid:solvent ratio of 1:10), homogenized using ULTRA-TURRAX (IKA, model T18, Germany) for 1 min at 10,000 rpm, sonicated for 10 min in a sonicator bath (100 W; 50 Hz; Ultrasons, JP Selecta™, Barcelona, Spain), and centrifuged (10 min; 4°C; 7,200 *g*). The supernatant was then collected. The extraction was repeated with aqueous acetone (30:70 v/v) instead of aqueous methanol. Both supernatants were mixed and evaporated under a vacuum. The extract was resuspended in 10–15 mL of water and passed under the C18 solid phase extraction cartridge (small (0.4 mL) (CHROMAFIX^®^)) to be concentrated. The final extract was eluted in a formic acid:methanol solution for HPLC (1:99; v/v).

#### 2.2.2 Insoluble-bound (poly)phenols

The pellet obtained in Section 2.2.1 was hydrolyzed with 40 mL of NaOH (4 M) for 4 h in an agitation bath (100 rpm) and darkness. After the incubation period, the sample was acidified using HCl (6 M) to achieve pH 2.0. Then, the sample was centrifuged (20 min; 4°C; 7,200 *g*). The supernatant was collected in a separatory funnel and mixed with ethyl acetate (30 mL). The funnel was manually agitated for 2 min and left overnight. When the phases were separated, ethyl acetate was collected, and the aqueous phase was cleaned two times with 25 mL of ethyl acetate each time. Ethyl acetate was evaporated under a vacuum. The extract was resuspended in 5 mL of water and concentrated using a C18 solid phase extraction cartridge (0.4 mL) (CHROMAFIX^®^). The final extract was eluted in a formic acid:methanol solution for HPLC (1:99; v/v).

#### 2.2.3 Soluble-conjugated (poly)phenols

Soluble-conjugate (poly)phenols were extracted following the methodology reported by Wang et al. (2016) with slight modifications. In brief, the soluble-conjugate (poly)phenols were extracted over the aqueous extract obtained in Section 2.2.1. A significant difference from the method described by Wang et al. (2016) was the use of methanol and acetone instead of ethanol. The aqueous extract underwent the same extraction process described in Section 2.2.2.

#### 2.2.4 Ultrasound-assisted extraction

The UP400St ultrasonic processor (400 W; 24 kHz) (Hielscher Ultrasonics GmbH, Germany) coupled to a sonotrode (S24d7 or S24d14D) was used to carry out ultrasound-assisted extraction. Eight extractions were performed under specific conditions (Table 1). An ice bath was used during ultrasonic treatment to prevent overheating. After ultrasonic treatment, the samples were centrifuged (5 min; 4°C; 7,200 *g*). The collected supernatant was filtered in a vacuum, evaporated in a rotavapor (Büchi Rotavapor R-200; Büchi Labortechnik AG, Switzerland), and resuspended in 5–10 mL of water. The extract was concentrated using a C18 solid phase extraction cartridge (0.4 mL) (CHROMAFIX^®^) and eluted in a formic acid:methanol solution for HPLC (1:99; v/v).

**TABLE 1:** Plackett–Burman design (PBD) and hydroxycinnamic acids, flavan-3-ols, flavonols, and total (poly)phenols (sum of detected compounds) from date palm seed flour using different levels of PBD extraction factors.

Run	Factors							Response					
	*X*_*1*_	*X*_*2*_ (%)	*X*_*3*_	*X*_*4*_ (mL/g)	*X*_*5*_	*X*_*6*_ (min)	*X*_*7*_ (%)	HA (mg/100 g)	Flavan-3-ols (mg/100 g)	Flavonols (mg/100 g)	Total (poly)phenols (mg/100 g)	FRAP (mg TE/g)	DPPH (mg TE/g)
1	1^a^	80	2	200	1	12	50	14.20 ± 0.09	86.71 ± 7.41	14.71 ± 0.54	115.62 ± 7.87	9.75 ± 0.65	3.14 ± 0.04
2	1	0	7	100	1	12	100	3.24 ± 0.11	37.84 ± 0.85	3.81 ± 0.03	44.88 ± 0.70	3.33 ± 0.03	1.33 ± 0.00
3	1	80	2	100	2	2	100	6.72 ± 0.15	55.39 ± 0.64	10.39 ± 0.43	72.49 ± 0.06	9.73 ± 0.51	3.09 ± 0.12
4	1	0	7	200	2	2	50	9.98 ± 0.08	134.89 ± 0.23	9.02 ± 0.02	153.89 ± 0.29	12.77 ± 0.3	3.88 ± 0.06
5	2^b^	0	2	200	1	2	100	19.21 ± 0.05	130.46 ± 1.72	14.36 ± 0.31	164.04 ± 2.08	15.46 ± 0.81	4.57 ± 0.02
6	2	80	7	100	1	2	50	9.00 ± 0.01	94.90 ± 0.57	6.99 ± 0.23	110.89 ± 0.79	8.91 ± 0.01	2.40 ± 0.01
7	2	0	2	100	2	12	50	14.78 ± 0.29	96.69 ± 0.76	11.40 ± 0.25	122.86 ± 1.30	10.10 ± 1.14	2.53 ± 0.00
8	2	80	7	200	2	12	100	12.83 ± 0.17	163.30 ± 2.88	12.87 ± 0.03	189.00 ± 3.08	16.10 ± 0.51	4.67 ± 0.15

^a^ S24d14D ( ∅22mm ; 99 μm; 150–160 W).

^b^ S24d7 ( ∅7mm ; 164 μm; 70–80 W).

X_1_, ultrasound probe; X_2_, ethanol concentration; X_3_, pH value; X_4_, liquid:solid ratio; X_5_, extraction cycle; X_6_, extraction time; X_7_, ultrasound amplitude.

HA, hydroxycinnamic acid; TE, Trolox equivalent.

### 2.3 Identification and quantification of (poly)phenols

An HPLC instrument (Hewlett-Packard HPLC 1200 series 1200) coupled to a C18 column, Mediterranean Sea 18 (Teknokroma, Barcelona, Spain) (25 × 0.4 cm; 5 μm) was used to detect and quantify the (poly)phenol compounds. HPLC work conditions were the same as those used by Lucas-González et al. (2023). Acetonitrile and formic acid (1%) were set as the mobile phase, with gradient elution (increasing acetonitrile): 1 mL/min as elution gradient and 20 μL for injection volume. Wavelengths of 280, 325, 360, and 520 nm were selected to detect compounds. The (poly)phenols in the samples were identified by comparing their retention time and absorbance spectrum with the available standard. A standard curve of 5–7 points of the available pattern, eluted under the same conditions as the samples, was utilized for quantification. When a detected compound showed the same absorbance spectrum of a (poly)phenol subclass, but there was no standard, the available standard of that (poly)phenol subclass was used for quantification. For example, the standard curve of catechin was used to quantify tentatively and identify procyanidins. The caffeoyl shikimic acids were quantified with the standard curve of caffeic acid, and the glycosylated quercetins were quantified with the calibration curve of quercetin-3-rutinoside.

### 2.4 Antioxidant activity

Two *in vitro* methods were used to determine the antioxidant activity of the different studied extracts. The ferric ion reducing antioxidant power (FRAP) method and the 2,2ʹ-diphenyl-1-picrylhydrazyl radical scavenging method (DPPH) were used to detect the antioxidant activity. Both assays were carried out following the methodology previously described by Lucas-González et al. (2018) and utilized a standard curve of the Trolox reagent (1 mM mother solution).

#### 2.4.1 FRAP assay

The extracts (250 µL) were mixed with phosphate buffer (0.2 M, pH 6.6) and potassium ferricyanide (1%) (625 µL from each one) and incubated at 50°C for 20 min. After incubation, trichloroacetic acid (10%) (625 µL) was added to the samples and vortexed. Then, aliquots of the before samples (625 µL) were mixed with distilled water (625 µL) and iron trichloride (0.1%) (125 µL). After 10 min of reaction, the absorbance was measured at 700 nm. The blank was prepared by replacing the sample with distilled water. The results were expressed as mg Trolox equivalent (TE)/g sample.

#### 2.4.2 DPPH assay

The sample (0.2 mL) was mixed with 2 mL of DPPH solution (60 mM) and incubated in darkness for 15 min. Then, absorbance was measured at 517 nm. The results were expressed as mg TE/g sample.

### 2.5 Plackett–Burman design

The PBD was used to identify the most critical variables of (poly)phenol extraction from date palm seeds. Seven factors were studied: ethanol concentration, liquid:solid ratio (mL/g), kind of sonotrode, amplitude (%), extraction time, extractant pH, and extraction cycle. The studied factors were selected based on preliminary works in our laboratory (data not shown) and consulting bibliography (Ciğeroğlu et al., 2018; Chen et al., 2020; Anaya-Esparza et al., 2023). The studied runs are given in Table 1. A total of eight runs were carried out. Statistica v8.0 (StatSoft Inc., Tulsa, OK, United States) was used to generate and analyze the PBD.

### 2.6 Statistical analysis

The results are expressed as the mean ± standard deviation of two replicates. Statistica v8.0 (StatSoft Inc., Tulsa, OK, United States) was used to analyze the results and calculate the regression coefficients. SPSS (IBM SPSS Statistics version 26) was used to carry out an ANOVA (one-way) assay, and Pearson’s correlation analysis with a 95% confidence level was used to determine any significant differences (*p* < 0.05).

## 3 Results and discussion

### 3.1 (Poly)phenol profile of date palm seed

(Poly)phenols in plant cells are found in different forms: free, soluble-conjugated, and insoluble-bound forms. Free (poly)phenols are easily extracted from the plant material, whereas soluble-conjugated and insoluble-bound (poly)phenols require specific extraction techniques, such as hydrolysis and acidification, to release them. For example, bound (poly)phenols are covalently bound to cell wall components, such as cellulose and pectin. Determining all (poly)phenol fractions in foods is vital for optimizing extraction processes with emerging technologies and evaluating their efficiency in recovering (poly)phenols from plant material.

The (poly)phenol profile of date palm seed flour in the free, soluble-conjugate, and insoluble-bound fractions is shown in Table 2. Twenty-three compounds were quantified, that is, 6 phenolic acids and 17 flavonoids, which belong to flavanols and flavonol subclasses. The main compounds were protocatechuic acid and catechin, which represented around 62% of the amount of the total (poly)phenols, followed by *p*-coumaric acid, epicatechin, and ferulic acid. Although other authors have reported the main (poly)phenol to be catechin, benzoic acid, or *p*-coumaric (Bouhlali et al., 2020; Ranasinghe et al., 2023; Muñoz-Tebar et al., 2024), the profile is in agreement with previous reports on date palm (Ghnimi et al., 2017). Moreover, the abundance of flavan-3-ols has been previously reported by Djaoudene et al. (2021) in different Algerian cultivars of date seeds (*P. dactylifera* L.), who reported four procyanidins (trimeric and dimeric and B1, B2, and B2 3-O-gallate), and Bettaieb et al., 2023, who detected two dimeric and three trimeric procyanidins. Hilary et al. (2020) also detected five procyanidins, mainly dimers and trimers of type B, and different classes of caffeoyl shikimic acids and glycosylated quercetins in date seeds of the Khalas variety. These differences from previous works can be due to the different extraction methods and studied cultivars. For example, the catechin content in the present work was around 1.5- and 3.8-fold of that reported by other authors (Bettaieb et al., 2023; Ranasinghe et al., 2023; Muñoz-Tebar et al., 2024). However, generally, authors reported (poly)phenols of free fraction and rarely in insoluble-bound fraction. To the best of our knowledge, this is the first time that three (poly)phenol fractions of date seed have been studied. Nevertheless, other authors have studied the free fraction and the depolymerized fractions of (poly)phenols in date seed (Khalas variety): Habib et al. (2014) and Hilary et al. (2020). In both studies, an increase in catechin and epicatechin was reported after phloroglucinolysis due to the breakdown of procyanidins.

**TABLE 2:** (Poly)phenol profile (mg/100 g) and antioxidant activity (mg TE/g) of date palm seed flour in each studied fraction after conventional extraction methods.

		Free	Soluble conjugate	Insoluble-bound
Hydroxybenzoic acids	Protocatechuic acid*	nd	13.67 ± 1.87^b^	79.59 ± 4.81^a^
Hydroxycinnamic acids	Caffeic acid*	2.40 ± 0.12^b^	8.08 ± 2.18^a^	nd
	5-*O*-Caffeoyl shikimic acid	3.60 ± 0.04^a^	nd	nd
	4-*O*-Caffeoyl shikimic acid	1.55 ± 0.01^a^	nd	nd
	*p*-Coumaric acid*	0.31 ± 0.02^b^	10.70 ± 1.53^b^	31.16 ± 5.41^a^
	Ferulic acid*	0.29 ± 0.02^c^	12.25 ± 0.15^a^	7.46 ± 0.74^b^
Flavan-3-ols	Procyanidin 1	26.52 ± 2.58^a^	4.90 ± 0.88^b^	nd
	Procyanidin 2	7.97 ± 0.39^a^	1.47 ± 0.26^b^	nd
	Procyanidin 3	0.85 ± 0.19^b^	10.26 ± 1.63^a^	nd
	Procyanidin 4	3.77 ± 0.25^a^	4.84 ± 0.55^a^	nd
	Catechin*	19.30 ± 2.16^b^	58.38 ± 4.63^a^	18.96 ± 2.14^b^
	Procyanidin 5	6.76 ± 0.83^a^	7.24 ± 1.39^a^	nd
	Procyanidin 6	7.38 ± 0.95^ab^	1.86 ± 0.23^b^	9.59 ± 2.03^a^
	Epicatechin*	22.51 ± 2.55^a^	5.46 ± 1.63^b^	7.58 ± 0.97^b^
	Epigallocatechin-3-gallate*	2.42 ± 0.54^a^	2.93 ± 0.06^a^	nd
	Gallocatechin-3-gallate*	0.73 ± 0.04^a^	1.26 ± 0.2^a^	1.31 ± 0.1^a^
Flavonols	Quercetin 3-rutinoside*	0.28 ± 0.03^a^	nd	nd
	Quercetin 3-β-D-glucoside*	0.91 ± 0.08^a^	nd	nd
	Quercetin 3-rhamnoside*	3.55 ± 0.34^a^	nd	nd
	Quercetin glycosyde 4	1.37 ± 0.11^a^	nd	nd
	Quercetin glycosyde 5	0.34 ± 0.03^a^	nd	nd
	Quercetin glycosyde 6	0.13 ± 0.01^a^	nd	nd
	Quercetin*	0.16 ± 0.02^b^	1.18 ± 0.10^a^	0.33 ± 0.00^b^
Sum of sub-classes of compounds	Hydroxycinnamic acids	8.15 ± 0.20^b^	31.03 ± 3.86^a^	39.13 ± 5.6^a^
	Flavan-3-ols	96.93 ± 5.56^a^	95.24 ± 10.30^a^	27.06 ± 11.5^b^
	Flavanols	6.42 ± 0.59^a^	1.12 ± 0.05^b^	0.33 ± 0.00^b^
Total		111.51 ± 5.17^a^	127.39 ± 14.12^a^	66.51 ± 17.10^a^
Antioxidant activity	FRAP	8.56 ± 1.01^b^	13.10 ± 0.27^a^	9.02 ± 1.81^a^
	DPPH	2.01 ± 0.23^c^	3.99 ± 0.19^a^	3.17 ± 0.10^b^

*Compounds confirmed by standard.

nd, not detected.

TE, Trolox equivalent.

Values with different letters within the same row indicate significant differences (*p* < 0.05) according to Tukey’s multiple range test.

Regarding the distribution of (poly)phenols in date palm seed, glycosylated quercetins and caffeoyl shikimic acids were only detected in the free fraction, whereas quercetin and caffeic acid were more abundant in the soluble-conjugate fraction than in free forms (*p* < 0.05). Moreover, the sum of the quantified flavan-3-ols showed a similar amount between free and soluble-conjugate fractions (*p* > 0.05), but the distribution was dependent on the compound. For example, catechin and procyanidin 3 were present in the highest amount in the soluble-conjugate forms, whereas epicatechin and procyanidin 1 showed their maximum concentration in the free fraction. These results were contrary to those reported by Sirisena et al. (2017), who found more epicatechin in the insoluble-bound form and did not report insoluble-bound catechin. This discrepancy could be due to the different extraction methods used to achieve (poly)phenols in the bound fraction and the cultivar (Deglet Nour vs Medjool). As can be expected, in the insoluble-bound fraction, the phenolic acids were the most abundant, followed by flavan-3-ols and quercetin with the lowest amount. Both protocatechuic acid and *p*-coumaric acid exhibited highest concentrations in their bound forms (*p* < 0.05). However, ferulic acid was present in the highest amount in the soluble-conjugate form.

### 3.2 Antioxidant activity

The antioxidant potential of date palm seed extracts obtained through conventional methods and UAE was studied using two different *in vitro* antioxidant assays, namely, DPPH and FRAP. As shown in Table 2, the values reported were slightly higher than those reported by Muñoz-Tebar et al. (2024) and lower than those reported by Bettaieb et al. (2023). Compared with other vegetable sources with similar particle sizes and whose antioxidant activity was measured following the same protocols, the antioxidant activity of date palm seed extracts was higher than that of persimmon flour and quinoa (Lucas-González et al., 2018; Pellegrini et al., 2018) but lower than that of cacao shell, especially the antioxidant activity determined using the ABTS method (Botella-Martínez et al., 2021; Delgado-Ospina et al., 2021).

Among the various (poly)phenol fractions studied, the highest antioxidant activity (as determined by FRAP and ABTS assays) was observed in the soluble-conjugate fraction, followed by the insoluble-bound and free fractions (*p* < 0.05). Interestingly, the total amount of (poly)phenols was similar in each studied fraction; however, the bound fraction exhibited the lowest values. However, no significant statistical differences were reported (Table 2). These results indicated that the kind of (poly)phenols in date palm seed extracts influences their antioxidant activity. Using Pearson’s correlation analysis (Supplementary Table S1), among the three studied (poly)phenol subclasses, flavan-3-ols reported the highest positive correlations, with values being 0.77 and 0.66 (*p* < 0.001), in FRAP and ABTS assays, respectively. Both correlations were similar to those observed for the total (poly)phenol content, that is, 0.83 and 0.75 (*p* < 0.001), respectively. Furthermore, both catechin and procyanidin 5, both flavan-3-ols, reported significant positive correlations, which are higher than 0.67 for FRAP values (*p* < 0.001) and around 0.58–0.61 for ABTS values (*p* < 0.01).

### 3.3 Plackett–Burman design

The response of the total (poly)phenols (sum of quantified (poly)phenols), hydroxycinnamic acids, flavan-3-ols, flavonols, and individual detected (poly)phenols after PBD is given in Table 1 and Supplementary Table S2. The global analysis of estimated effects and regression analysis are given in Supplementary Tables S3, S4.

**FIGURE 1:**
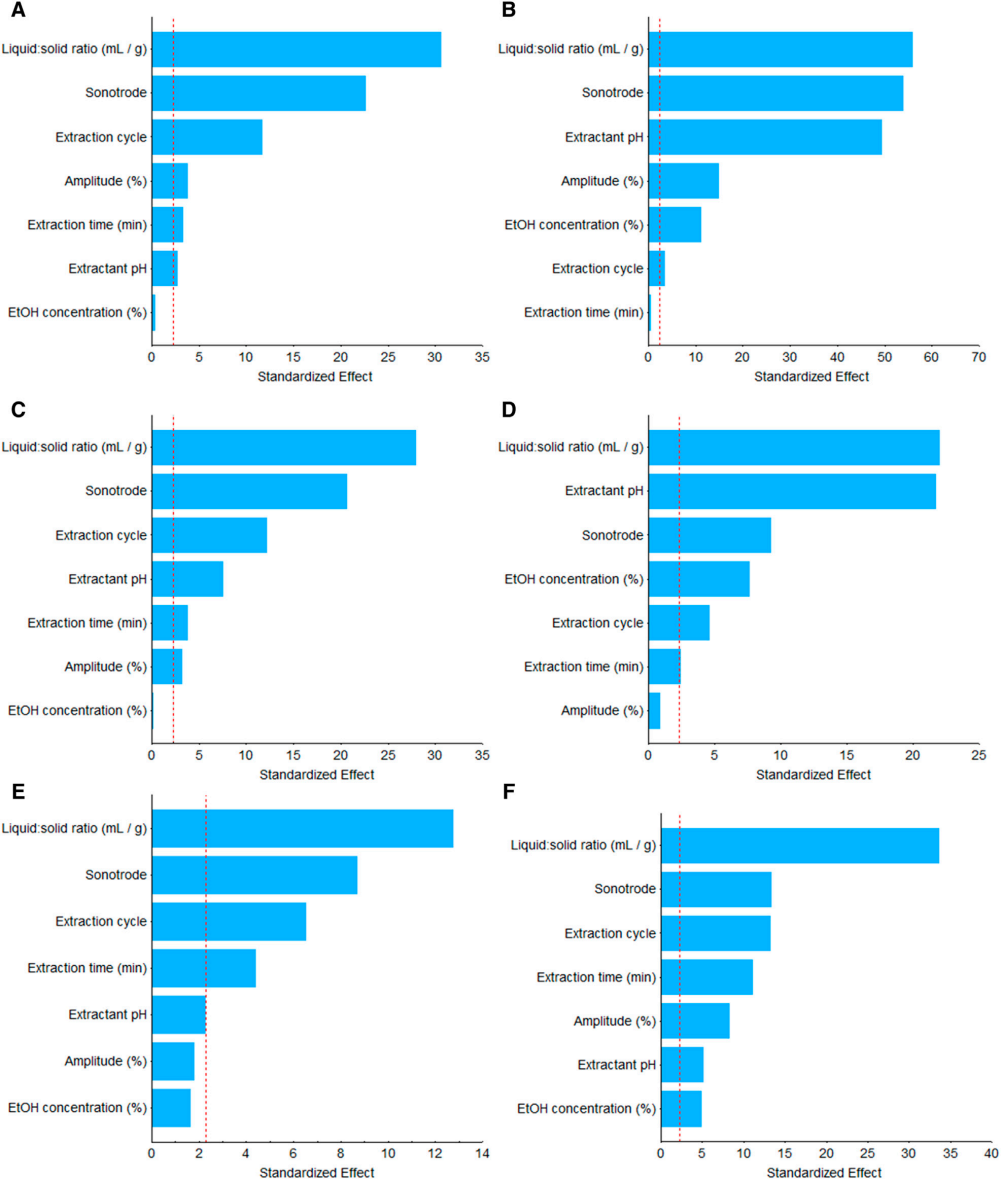
Pareto chart of standardized effect estimates of **(A)** total (poly)phenols, **(B)** hydroxycinnamic acids, **(C)** flavan-3-ols, and **(D)** flavonols, and antioxidant activity. **(E)** FRAP and **(F)** DPPH of palm date seed flour. Vertical line in the chart defines the 95% confidence level.

The Pareto chart is a graphical representation in which the ANOVA effect estimates are sorted from the largest absolute value to the smallest absolute value, which was used to identify critical variables among the studied parameters (Figure 1; Supplementary Table S3). The red line indicates a 95% confidence level and highlights the limit of critical values (2.305) where parameters are considered significant. Furthermore, the statistical significance of the studied variables was classified as extremely significant, very significant, and significant when *p*-values were <0.001, <0.01, and < 0.05, respectively (Supplementary Tables S3, S4).

Regarding total (poly)phenol recovery after UAE from date palm seed, significant responses were observed for six factors, which were arranged in decreasing order based on their estimated standardized effects as follows: liquid:solid ratio, sonotrode, extraction cycle, amplitude, extraction time, and extractant pH (Figure 1A). The only studied factor that did not influence, in a significant manner, the total (poly)phenol extraction was ethanol concentration (*p* > 0.05). The liquid:solid ratio was also the most critical factor in the UAE for the recovery of total flavan-3-ols, flavanols, and hydroxycinnamic acids, as well as for their antioxidant activity, showing in all cases an extremely significant response (*p* < 0.001) and positive effect (Figure 1). These results may be due to an increase in the viscosity of the extraction medium when the liquid:solid ratio was lower. Muñoz-Tebar et al. (2024) reported that date seed flour with a particle size smaller than 0.210 mm contains a total dietary fiber amount of 67.89% and its swelling capacity was approximately 25 mL/g. Viscosity is considered the most significant thermodynamic property that affects sonoprocessing properties. The increase in the viscosity of the medium causes a decrease in the volume of active regions and in the magnitude of the acoustic streaming field (Schieppati et al., 2024), which causes the power at which the sonotrode works to increase, and the mass transference decreases (Huang et al., 2024). Therefore, ultrasound extraction could facilitate the release of polysaccharides, modifying the medium viscosity and reducing the effect of cavitation bubbles. Another possibility was the saturation of the extractant due to the release of polysaccharide compounds into the medium. However, further analysis is required to gain a better understanding of the impact and interactions of the extraction conditions of (poly)phenols from date palm seed.

The other critical variable that affected the (poly)phenol extractions was the kind of sonotrode (Figure 1). Previously, many authors reported that the sonotrode tip shape affects acoustic streaming (Fang et al., 2018). Even the probe immersion depth affects sonoprocessing (increased immersion is related to a larger active region volume and negative acoustic pressure at low viscosity) (Schieppati et al., 2024). The sonotrode S24d7, which has a lower diameter and energy input but higher displacement amplitude than the other studied probe (S24d22D), showed the best extraction recovery. In future works, the relation between the sonotrode and amplitude must be considered.

As previously remarked, the liquid:solid ratio and the kind of sonotrode were the most critical factors for the recovery of total (poly)phenols, hydroxycinnamic acids, flavan-3-ols, and flavonols from date palm seed flour. Regarding the other studied factors, their impact on the compound recovery depended on the nature of the (poly)phenols. For example, the extraction cycle was an extremely significant factor in the recovery of flavan-3-ols but reported an inferior significance (*p* < 0.05) in the recovery of hydroxycinnamic acids (Figures 1B, C; Supplementary Table S3). Similarly, the extractant pH and the ethanol concentration were critical factors in the extraction of hydroxycinnamic acids and flavonols but not in the recovery of flava-3-ols (*p* > 0.05). Moreover, among compounds within the same sub-classes, differences can be observed. For example, in the recovery of catechin, the ethanol concentration was an insignificant factor, whereas for the recovery of gallocatechin-3-gallate, it was the most positive and extremely significant factor (Supplementary Table S3). Similar is the case for quercetin and glycosylated quercetins. For the recovery of quercetin, the ethanol concentration was the most crucial and positive factor, whereas for quercetin 3-rhamnoside, quercetin 3-β-D-glucoside, and quercetin 3-rutinoside, the liquid:solid ratio had a higher effect than the ethanol concentration (Supplementary Table S3).

Concerning the effect of the studied factors on the antioxidant activity of extracts, in the case of FRAP, the results were very close to those shown by flavan-3-ols (Figures 1C, E). As previously mentioned, a positive and significant correlation between the amount of flavan-3-ols and the reducing antioxidant power of samples was observed (Supplementary Table S3). In the case of the ability of the sample to neutralize free radicals, the seven factors were significant (*p* < 0.05) (Figure 1F; Supplementary Table S3). The three studied factors with the highest estimated effects were the same as those reported for flavan-3-ols. A positive and significant correlation between the content of flavan-3-ols and DPPH values in the samples was also shown, but it was lower than that reported by FRAP values (Supplementary Table S1). Therefore, the reported results were consistent.

### 3.4 Conventional extraction methods vs. ultrasound-assisted extraction

Phytochemical extraction processes are widely used in the food, pharmaceutical, and cosmetic industries. These processes typically involve using organic solvents, which are often volatile, derived from petroleum, and harmful to health. Moreover, they are time-consuming and require high temperatures (40–100°C) (Rifna et al., 2023). The current trend is to reduce the dependence on organic solvents by utilizing emerging technologies and environmentally friendly extractants such as water or ethanol, which are generally recognized as safe (GRAS) and can be obtained through biomass processing (Chemat et al., 2019).

UAE is an emerging technology that uses an acoustic cavitation phenomenon to disrupt vegetable tissues and promote the release of phytochemical compounds (Martínez-Olivo et al., 2023). This technique can be implemented using an ultrasound bath or a probe. Despite both methods utilizing cavitation, there are significant differences between them. Probe-type sonication exerts higher intensity than ultrasound baths and is easily industrial-scalable. Aznar-Ramos et al. (2022) reported higher recovery in the extraction of (poly)phenols from mango peels through UAE coupled to a probe than using an ultrasound bath. Similar results have been observed in the present work. The effectiveness to extract free (poly)phenols such as caffeoylquinic acids and glycosylated quercetins was improved using probe-type sonication. Both caffeoylquinic acids and glycosylated quercetins, which were only observed in the free fraction, were observed in the highest amount after probe-type sonication (*p* < 0.05) (Table 3). For example, the content of quercetin-3-rutinoside increased 3.5-fold and that of 5-O-caffeoylshikimic acid increased 1.5-fold after probe-type sonication. These results highlight that it is possible to reduce the use of organic solvents, such as methanol and acetone, for the extraction of free polyphenols in date palm seed.

**TABLE 3:** Total (poly)phenol profile of date palm seed flour after conventional extraction (sum of free, soluble-conjugate, and insoluble-bound fractions) and UAE (mg/100 g).

		Conventional extraction	UAE	Sig.	Effect	Run^¥^
Hydroxybenzoic acids	Protocatechuic acid*	87.36 ± 12.58	nd	na	↓	
Hydroxycinnamic acids	Ferulic acid*	20.51 ± 0.03	0.58 ± 0.01	**	↓	7
	Caffeic acid*	9.22 ± 1.04	5.69 ± 0.00	ns	↓	5
	5-*O*-Caffeoyl shikimic acid	3.60 ± 0.04	9.12 ± 0.06	***	↑	5
	4-*O*-Caffeoyl shikimic acid	1.55 ± 0.01	3.38 ± 0.01	***	↑	5
	*p*-Coumaric acid*	41.99 ± 4.02	0.51 ± 0.07	**	↓	8
Flavan-3-ols	Catechin*	75.35 ± 12.35	37.19 ± 0.83	*	↓	8
	Procyanidin 1	28.53 ± 4.59	45.25 ± 0.35	*	↑	8
	Procyanidin 2	8.57 ± 0.22	11.96 ± 0.64	ns	=	8
	Procyanidins 3	11.11 ± 1.44	5.15 ± 0.87	ns	=	1
	Procyanidin 4	6.60 ± 2.31	7.66 ± 0.20	ns	=	8
	Epicatechin*	33.23 ± 2.89	33.23 ± 5.38	ns	=	1
	Procyanidin 5	10.20 ± 1.57	9.80 ± 0.42	ns	=	8
	Procyanidin 6	14.66 ± 5.02	12.13 ± 0.45	*	↑	5
	Epigallocatechin-3-gallate*	4.41 ± 1.41	4.63 ± 0.04	ns	=	8
	Gallocatechin-3-gallate*	2.91 ± 0.65	0.73 ± 0.09	*	↓	1
Flavonols	Quercetin 3-rutinoside*	0.30 ± 0.01	1.36 ± 0.01	***	↑	4
	Quercetin 3-β-D-glucoside*	0.97 ± 0.03	2.26 ± 0.15	*	↑	1
	Quercetin 3-rhamnoside*	1.56 ± 0.08	0.27 ± 0.01	**	↓	1
	Quercetin glycoside 4	1.37 ± 0.11	3.05 ± 0.15	*	↑	1
	Quercetin glycoside 5	0.34 ± 0.03	0.78 ± 0.05	*	↑	5
	Quercetin glycoside 6	0.13 ± 0.01	0.41 ± 0.01	**	↑	8
	Quercetin	3.78 ± 0.09	7.74 ± 0.53	*	↑	1
Sum of family compounds	Hydroxycinnamic acids	74.28 ± 5.56	19.21 ± 0.05	*	↓	5
	Flavan-3-ols	177.50 ± 37.38	163.3 ± 2.88	ns	=	8
	Flavanols	7.91 ± 0.60	14.71 ± 0.54	*	↑	1
Total		259.69 ± 43.54	189.00 ± 3.08	ns	=	8

*Compounds confirmed by pattern.

^¥^Run where value was taken for comparing with conventional extraction methods.

*Significant difference, *p* < 0.05.

**Very significant difference, *p* < 0.01.

***Extremely significant difference, *p* < 0.001.

X_1_, ultrasound probe; X_2_, ethanol concentration; X_3_, pH value; X_4_, liquid:solid ratio; X_5_, extraction cycle; X_6_, extraction time; X_7_, ultrasound amplitude.

UAE, ultrasound-assisted extraction.

nd, not detected.

na, not applicable.

ns, not significant.

Opposite probe-type sonication was inefficient in extracting bound (poly)phenols, such as protocatechuic acid, *p*-coumaric acid, or ferulic acid (Table 3), which highlights that basic hydrolysis, followed by liquid–liquid extraction with ethyl acetate, showed better results than UAE. However, it seems that efficiency releases soluble-conjugate (poly)phenols. For example, no significant differences were shown between the total content of many flavan-3-ols, like some procyanidins (*p* < 0.05), between conventional extraction and UAE. Nevertheless, a pronounced reduction in the catechin content was observed, which reached its maximum concentration in the soluble-conjugate fraction (*p* < 0.05) (Tables 2, 3). Therefore, more research is needed to optimize the extraction of soluble-conjugate (poly)phenols through UAE.

## 4 Conclusion

In the current work, the efficiency of extracting (poly)phenols from date palm seeds using UAE (probe-type sonication) has been investigated. As a novelty, the profile of the (poly)phenols has been used to select critical factors in the UAE. Authors typically use the spectrophotometric method to optimize or study the most crucial factors that affect extraction methods, such as total phenol content, total flavonoid content, or anthocyanin content (Chebbi et al., 2024; Lee et al., 2024; Soukaina et al., 2024). However, investigating the effect of different variables on the (poly)phenol profile is relevant to maximize the extraction of target compounds in plant materials.

Twenty-three compounds were quantified with the conventional extraction methods, with protocatechuic acid and catechin being the predominant (poly)phenols. Using an ultrasonicator processer coupled to a sonotrode, 22 compounds were quantified. Probe-type sonication presented great efficiency in extracting free and soluble-conjugated (poly)phenols from date palm seed flour compared to conventional extraction. However, it was not efficient in extracting insoluble-bound (poly)phenols. This fact pointed out that the cavitation process did not affect the covalent bond between the cell wall and (poly)phenols. Studying the effect of UAE on insoluble-bound (poly)phenols was also a novelty as this fraction is not usually determined by the authors. Therefore, this work highlights the limitations of the UAE for extracting bound (poly)phenol compounds.

Concerning variables that affected (poly)phenol extractions more, the Plackett–Burman design highlights that these were liquid:solid ratio and the kind of sonotrode. Furthermore, the results of the current work demonstrate that the nature of (poly)phenols determines their extractability under different extraction conditions. For example, flavan-3-ols were easily extracted with water, whereas quercetin extraction was concentration dependent on ethanol.

In conclusion, using UAE (probe-type sonication), extracts rich in (poly)phenols and with antioxidant activity derived from date palm seeds can be obtained in a short time and with eco-friendly solvents (water or ethanol). Even if the extraction target is the flavan-3-ols, water is an efficient extractant. In addition, probe-type sonication is a technique easily scalable to industrial processes. Thus, using water as a solvent, an antioxidant extract rich in flavan-3-ols and glycosylated-quercetins can be obtained, which could be used, for example, as a natural antioxidant in the food industry, promoting the valorization of date palm seed and circular economy. Nevertheless, more studies are needed to understand the effect of cavitation on the different fractions of (poly)phenols. Future research could focus on combining technologies, such as an enzymatic process coupled to UAE, to completely extract (poly)phenols from date palm seed.

## Data Availability

The original contributions presented in the study are included in the article/Supplementary Material: further inquiries can be directed to the corresponding author.
